# Radiation Exposure of Ovarian Cancer Patients: Contribution of CT Examinations Performed on Different MDCT (16 and 64 Slices) Scanners and Image Quality Evaluation: An Observational Study

**DOI:** 10.1097/MD.0000000000000765

**Published:** 2015-05-01

**Authors:** Stefania Rizzo, Daniela Origgi, Sarah Brambilla, Federica De Maria, Riccardo Foà, Sara Raimondi, Nicoletta Colombo, Massimo Bellomi

**Affiliations:** From the Department of Radiology, European Institute of Oncology, via Ripamonti 435, 20141 Milan (SR, MB), Department of Medical Physics, European Institute of Oncology, via Ripamonti 435, 20141 Milan (DO); Department of Health Sciences, University of Milan, via A.di Rudinì 8, 20142 Milan (SB, FDM, RF, MB); Department of Epidemiology and Biostatistics, European Institute of Oncology, via Ripamonti 435, 20141 Milan (SR); and Department of Gynecology, European Institute of Oncology, via Ripamonti 435, 20141 Milan, Italy (NC).

## Abstract

The objective of this study is to compare radiation doses given to ovarian cancer patients by different computed tomographies (CTs) and to evaluate association between doses and subjective and objective image quality.

CT examinations included were performed either on a 16-slice CT, equipped with automatic *z*-axis tube current modulation, or on a 64-slice CT, equipped with *z*-axis, *xy*-axis modulation, and adaptive statistical iterative algorithm (ASIR). Evaluation of dose included the following dose descriptors: volumetric CT dose index (CTDI_vol_), dose length product (DLP), and effective dose (E). Objective image noise was evaluated in abdominal aorta and liver. Subjective image quality was evaluated by assessment of image noise, spatial resolution and diagnostic acceptability.

Mean and median CTDI_vol_, DLP, and E; correlation between CTDI_vol_ and DLP and patients’ weight; comparison of objective noise for the 2 scanners; association between dose descriptors and subjective image quality.

The 64-slice CT delivered to patients 24.5% lower dose (*P* < 0.0001) than 16-slice CT. There was a significant correlation between all dose descriptors (CTDI_vol,_ DLP, E) and weight (*P* < 0.0001). Objective noise was comparable for the 2 CT scanners. There was a significant correlation between dose descriptors and image noise for the 64-slice CT, and between dose descriptors and spatial resolution for the 16-slice CT.

Current dose reduction systems may reduce radiation dose without significantly affecting image quality and diagnostic acceptability of CT exams.

## Introduction

Ovarian cancer is the fifth leading cancer type of death, with 22,240 estimated new cases and 14,030 estimated deaths in 2013 in the US.^1^ Thanking the advances in treatment options, the 5-year survival rate has been progressively increasing,^2^ with a 92% 5-year survival rate for patients with stage I at diagnosis.^3^

Multi-detector computed tomography (MDCT) is currently considered the best imaging technique for staging and follow-up of ovarian cancer patients. Computed tomography (CT) is indeed widely available and the required information is provided in a relatively short examination time.^4^ During follow-up, ovarian cancer patients undergo CT examinations at least once a year, therefore methods to reduce or modulate the radiation dose have to be considered.

Automatic tube current modulation (ATCM) is based on the principle that X-ray attenuation and quantum image noise are determined by the size of the object and by its tissue density. Then, tube current can be adjusted by changing regional attenuation to maintain image quality and increase radiation dose efficiency.^5^

Since the introduction of MDCT in the late 1990s, filtered back projection (FBP) algorithms have been used for CT image reconstruction owing their faster image reconstruction and ease of implementation.^6^

Over the past decade, the desire for better resolution, greater volume coverage, and faster scan times, along with the need to lower radiation dose, have raised the need of alternative techniques. With this regard, adaptive statistical iterative reconstruction (ASIR) algorithm has become available for clinical use. This algorithm takes into account precise modeling of the X-ray photon statistics and electronic noise, and utilizes the information contained in the FBP-reconstructed image as an initial “building block” in the reconstruction process.^7^ To our knowledge, the role of ASIR in reducing radiation dose in CT scans of thorax abdomen and pelvis, performed during follow-up of ovarian cancer patients, has not been evaluated.

The objectives of this study were: to compare radiation dose given to ovarian cancer patients who underwent a follow-up CT scan either on a 16-slice CT machine, equipped with *z*-axis ATCM, or on a 64-slice CT machine, equipped with *xy*-modulation, *z*-axis modulation and with the ASIR algorithm; to compare objective and subjective image quality on CT images obtained; to evaluate association between radiation dose and image quality.

## METHODS

### Participants and CT Examinations Selection

For this cross-sectional study, all patients who underwent CT examinations for follow-up of ovarian cancer between January 2013 and September 2013, were retrospectively included. CT examinations extended to thorax, abdomen and pelvis (TAP), examinations limited to abdomen and pelvis were subsequently excluded in order to avoid bias related to the extension of the CT exam.

Date of the CT exam and age of the patients were recorded, along with weight (in kg) and, when present, with body mass index (BMI).

The institutional review board approved this observational retrospective study with waive of informed consent. Indeed, written informed consent to undergo the examination, as well as to the use of anonymized clinical and imaging data for scientific and/or educational purposes, had been obtained from all patients before undergoing the CT examination

### CT Acquisition

The CT examinations were performed either on a 16-slice Lightspeed CT scanner (General Electric Healthcare, Milwaukee WI, USA) or on a 64-slice GE MSTC Optima 660 (General Electric Healthcare, Milwaukee, WI). In order to avoid comparison of doses from examinations with multiple phase acquisition, all scans included extended in cranio-caudal direction from the apices of the lungs to the pubis, only in the portal venous phase (70–90 sec after the i.v. administration of contrast medium). All patients were asked to fast for at least 6 hours prior to the exam, and were given negative oral contrast (water) 30 minutes before the exam. During the period selected, the following contrast agents were randomly used: Visipaque^®^ 320 (GE Healthcare, Milan, IT), Iomeron^®^ 350 (Bracco, Milan, Italy). All the images were archived in digital format.

On the 16-slice CT scanner, scans were acquired during a single breath-hold at the following parameters: tube rotation time 0.8 second; pitch 1.75; standard soft-tissue algorithm reconstruction; collimation 20 mm (16 mm × 1.25 mm); slice thickness 2.5 mm; reconstruction interval 2.5 mm; display field of view (DFOV) 320 to 360 mm; tube voltage 120 kV; tube current 100 to 440 mA, according to the use of ATCM (auto-mA), with a noise index (NI) equal to 11.57.

On the 64-slice CT scanner, scans were acquired during a single breath-hold at the following parameters: tube rotation time 0.6 second; pitch 1.375; standard soft-tissue algorithm reconstruction; collimation 20 mm (32 mm × 0.625 mm); slice thickness 2.5 mm; reconstruction interval 2.5 mm; display field of view (DFOV) 320 to 360 mm; tube voltage 120 kV; tube current 80 to 440 mA according to the use of ATCM (auto-mA), with a NI = 18.2. The MDCT Optima 660 is also equipped with the ASIR algorithm that was selected at a 40% level of blending.

### CT Exposure: Evaluation of Dose

For each patient the following data were recorded on an Excel spreadsheet file (Microsoft Office Excel 2007, Richmond, VA): volume CT dose index (CTDI_vol_) and dose length product (DLP) as indicated on the final dose report; extension of each acquisition (in cm).

The CTDI_vol_, used by the American College of Radiology (ACR) for CT practice accreditation, indicates the estimated mean dose for a single slice. Its unit is milliGray (mGy).

The DLP, that is equal to the CTDI_vol_ multiplied by the scan length, is expressed in mGy × cm, and indicates the overall radiation absorbed by the patient. DLP is also an helpful indicator to estimate the effective dose (E) (mSv). E quantifies the overall risk induced by ionising radiation (fatal and nonfatal cancers induction) referring to a “standard” patient averaged over all ages and both sexes.^8^ Calculation of effective doses in CT requires appropriate effective dose per unit DLP (E/DLP) conversion factors.

The effective doses were calculated from the DLP, with the use of the updated weighting factors introduced by ICRP 103.^8,9^ In particular for thorax-abdomen and pelvis acquisitions, included in our study cohort, a weighting factor of 0.0186 mSv/mGyxcm was used.

### Objective Image Quality Assessment

Objective image noise (in Hounsfield units, HU) ± standard deviations and CT numbers (HU) were measured for all CT image series. Circular regions of interest (20–30 mm^2^) were drawn in the abdominal aorta, without touching the lumen walls, to cover at least two-thirds of its lumen. Circular regions of interest (20–30 mm^2^) were also drawn in a homogeneous area of the right lobe of the liver.^10^

### Subjective Image Quality Assessment

All randomized CT image data sets were reviewed at a picture archiving and communication systems diagnostic workstation, for assessment of subjective image quality. All image data sets were evaluated in consensus by 2 radiologists with 13 and 5 years of experience (SR, SB) for assessment of image quality of the whole exam, keeping in mind the clinical indication to perform the exam (follow-up of patient with ovarian cancer). Assessment of image quality was evaluated according to the general assessment of image quality attributes, described in the European Guidelines on Quality Criteria for Computerized Tomography document,^11^ used in multiple prior studies in the radiology literature.^10^ Acceptable noise and acceptable spatial resolution were graded on a 3 point scale (1 = too much; 2 = optimum; 3 = too little); diagnostic acceptability was graded on a 4 point scale (1 = fully acceptable; 2 = probably acceptable; 3 = only acceptable under limited conditions; 4 = unacceptable). When the diagnostic acceptability was graded as 4 (unacceptable), reasons were given.

### Statistical Analysis

Informative parameters for the distribution of CTDI_vol_ and DLP (dose descriptors) were calculated for each CT scanner, and their distributions were tested for normality by the Kolmogorov–Smirnov test. Since the dose descriptors resulted not normally distributed, the non-parametric Wilcoxon 2-independent sample test was used to compare dose descriptors distribution between the 64-slice and 16-slice CT scanners.

The correlation between dose descriptors and patients’ weight (and BMI, when available) was evaluated with the Spearman correlation coefficient.

In order to compare objective and subjective image quality parameters between the 2 CT scanners, the non-parametric Wilcoxon 2-independent sample test and Chi Square test were, respectively, performed. Among each CT scanner, the association between CT dose descriptors and subjective image quality descriptors was evaluated by the non-parametric Kruskal–Wallis test. To evaluate the association between objective and subjective image quality unrelated to the CT scanner used, Kruskal–Wallis test was used for the CT scans altogether.

Multivariate analysis was also performed to adjust results by patients weight. A multivariate analysis of variance (ANOVA) on log-transformed variables was performed to assess association of different CT scans with dose descriptors and objective noise. A weight-adjusted logistic regression analysis was performed to assess the association of different CT scans with subjective image quality parameters.

*P*-values < 0.05 were considered significant. Statistical analyses were performed with SAS software (version 9.2).

## RESULTS

### Participants and CT Examinations

From the initial group of patients, 37 were excluded because CT scan included only abdomen and pelvis (n = 24) or was performed as a multiple phase examination (n = 13).

Patients included were 297, mean age was 59 years (range 19–88). 149/297(50%) examinations were performed on the 16-slice Lightspeed CT scanner, 148/297(50%) were performed on the 64-slices MSTC Optima 660. The mean weight of the patients was 64 kg; BMI was available only for 71/297 patients, all of which underwent examination on the 64-slices scan. The mean age was comparable between the 2 groups of patients (58; range: 19–85 and 60; range: 27–88 for 16-slices and 64-slices CT, respectively, *P* = 0.47), while the mean weight was higher for 64-slices CT (66 kg; range: 39–110) than for 16-slices CT (61 kg; range: 40–85): *P* = 0.007.

### CT Exposure

**FIGURE 1:**
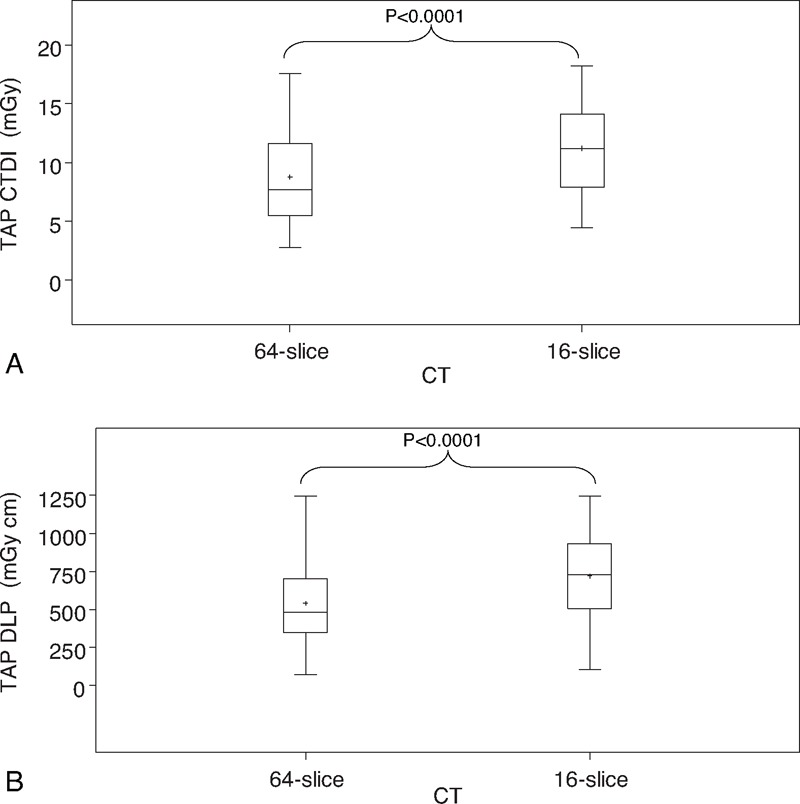
Box plot representing the (A) TAP CTDI_vol_ and (B) TAP DLP received by patients with 16-slice or 64-slice CT. Minimum and maximum are depicted by black whiskers, the box signifies the upper and lower quartiles, the median and the mean are represented, respectively by a black line and a small cross within the box.

Box plots for the distribution of CTDI_vol_ and DLP according to 64-slice and 16-slice scanner are shown in Figure 1. There was a significant difference of dose descriptors values between the 2 different CT scanners (*P* < 0.0001). Mean and median of E were 12.22 and 14.23 for the 16-slice Lightspeed CT scanner, and 9.23 and 6.86 for 64-slices MDCT Optima 660, respectively, with a lower mean for the 64-slices CT of about 24.5%. No change in *P*-values was observed after adjustment by patients weight (results not shown).

**FIGURE 2:**
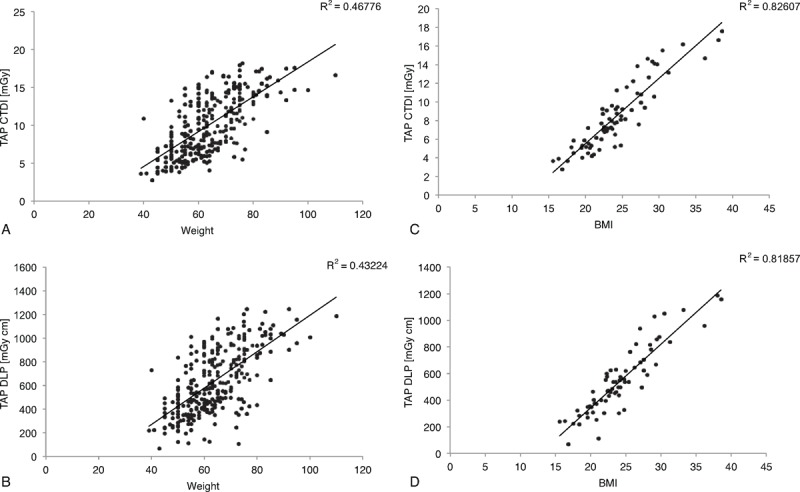
Correlation between (A) TAP CTDI and patients weight, (B) TAP DLP and patients weight, (C) TAP CTDI and patients BMI (N = 71), (D) TAP DLP and patients BMI (N = 71).

Correlation between CTDI_vol_ and DLP with all patients’ weight is shown in Figure 2. We observed a significant increase of dose descriptors along with weight increase (*P* < 0.0001), and with BMI for the 71 patients where BMI was available (*P* < 0.0001, figure not shown).

### Objective Image Quality

**FIGURE 3:**
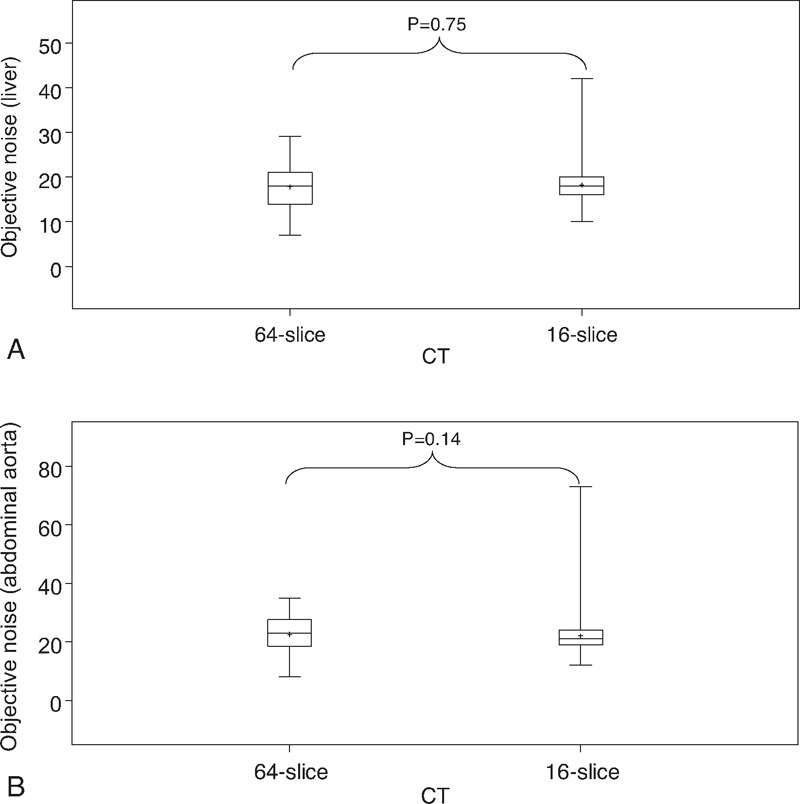
Box plot representing the total objective noise evaluated in (A) liver (B) abdominal aorta with 16-slice or 64-slice CT. Minimum and maximum are depicted by black whiskers, the box signifies the upper and lower quartiles, the median and the mean are represented, respectively by a black line and a small cross within the box.

Total objective noise evaluated in liver and abdominal aorta with 16-slice and 64-slice CT was not significantly different (*P* = 0.75 and *P* = 0.14, respectively) between the 2 scanners (Figure 3). The lack of significance was also observed after adjustment by patients weight (results not shown).

### Subjective Image Quality

Subjective image quality scores did not differ between the 2 groups of examinations performed on the 16-slice and 64-slice CT scanners (Table 1), even after adjustment by patients weight (results not shown).

**TABLE 1:**
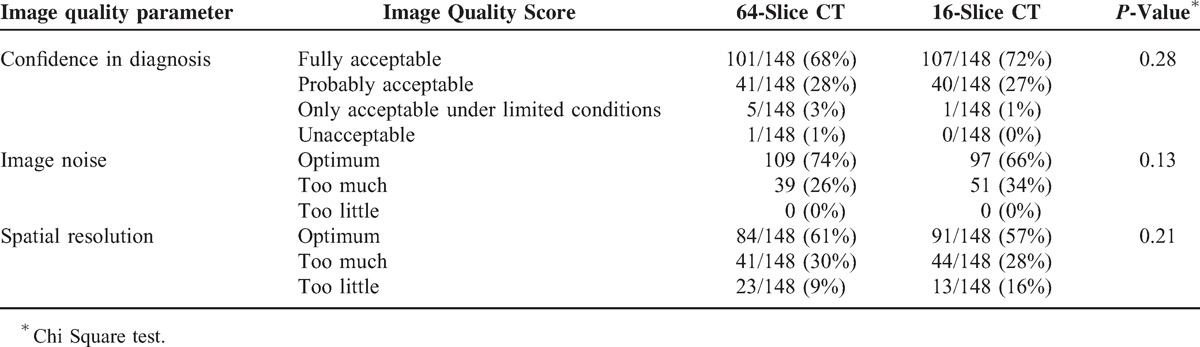
Subjective Image Quality Scores According to 16-Slice or 64-Slice CT

Looking at the 2 CT scanners separately, for the 64-slice CT a significant association between CT dose descriptors and subjective image noise assessment (*P* = 0.03 and *P* = 0.02, respectively) was shown, with higher noise for lower doses, and between CTDI_vol_ and spatial resolution, with optimum spatial resolution for higher doses (Table 2).

**TABLE 2:**
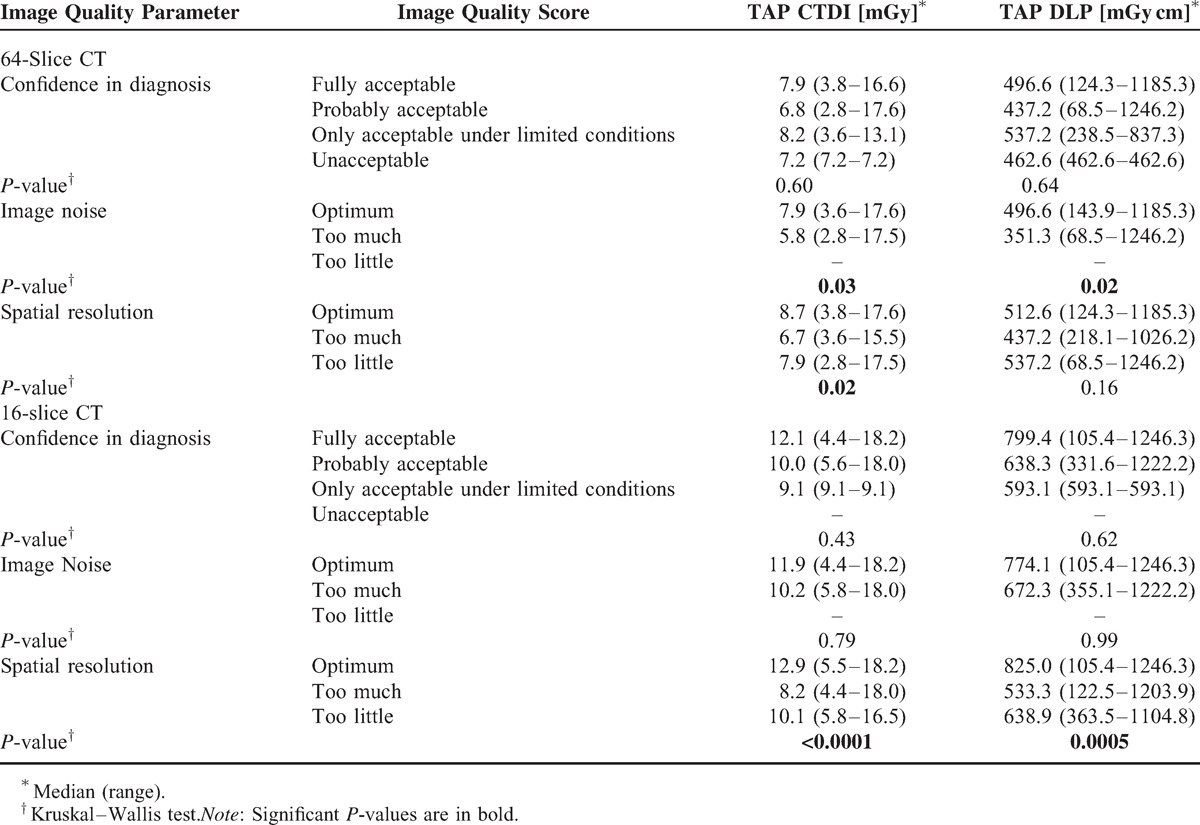
Association Between CT Dose and Subjective Image Quality Assessment Stratified by CT Scanner

For the 16-slice CT, a strong association between dose descriptors and spatial resolution (*P* < 0.0001 and *P* = 0.0002, respectively) was shown, with an optimum spatial resolution for higher doses.

Association between objective and subjective image quality assessment unrelated to the CT scanner is shown in Table 3. A higher confidence in diagnosis was reported for images with lower objective noise (*P* = 0.02).

**TABLE 3:**
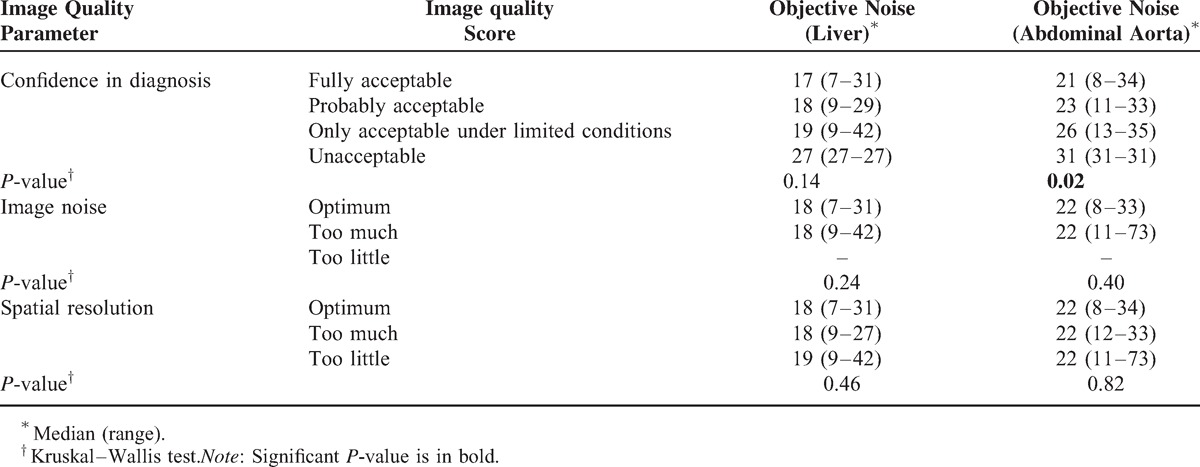
Association Between Objective and Subjective Image Quality Assessment for All the CT Examinations

## DISCUSSION

Current CT dose modulation techniques, as ASIR, may reduce radiation dose without significantly affecting image quality and diagnostic acceptability of the examinations.

Ovarian cancer patients are usually believed to have a short life expectancy and, consequently, radiation dose concerns are frequently overcome because the risk of a radio-induced tumour is considered less important than a good image quality of a CT scan. Nevertheless, advances in diagnostics and therapies of these patients have prolonged their life expectancy; indeed patients diagnosed in stage I of disease may have a 5-year survival rate of 92%.^3^ Therefore, it is important to make all of the efforts not to overexpose patients, especially if obtaining good quality images is possible maintaining radiation dose as low as reasonably possible (so-called ALARA principle).^12^

Known efforts to reduce radiation dose without compromising the quality of diagnostic information include: lowering the tube current–time product^13^; automatic exposure control^5^; reducing the peak kilovoltage^14^; and shielding radiosensitive organs.^15^

A technique to lower radiation dose, the reduction of tube current, is associated with significant increases in image noise when standard reconstruction method of FBP is used to obtain the final CT images. Iterative reconstruction (IR), routinely used for positron emission tomography (PET) and single photon emission computed tomography (SPECT), has been recently reintroduced to CT as an alternative mathematical algorithm that results in lower image noise than FBP.^16,17^ ASIR, a recently developed IR algorithm approved by the US Food and Drug Administration for clinical use, reduces reconstruction time by using information obtained from the FBP algorithm as a starting point for image reconstruction; then it repeatedly compares the estimated pixel value to the ideal value predicted by the noise model, until the estimated and ideal values converge.^17^

Multi-detector computed tomography (MDCT) is currently recommended by ESUR as the best imaging technique for staging and follow-up of ovarian cancer patients.^18^ CT examinations may vary greatly according to the number of phases acquired, but for follow-up examinations acquisition of a single portal venous phase is considered appropriate.^18^

In this study a significant difference in radiation dose was found, comparing 2 different MDCT scanners, equipped with different techniques of dose modulation. Specifically, the radiation dose given by the 64-slices Optima 660 scanner, equipped with the angular (*xy*) and longitudinal (*z*) modulation, along with the ASIR, gave to the patients a lower mean dose, of about 24.5%, than the 16-slice Lightspeed CT scanner, equipped with the longitudinal (*z*) ATCM. These data are supported by published data, demonstrating a reduction in radiation dose when ASIR is used,^7^ also for anatomical regions different from the abdomen,^19^ compared to other techniques of dose modulation.

As expected, we found a strongly significant correlation between dose descriptors and weight. Indeed, since the attenuation profile to modulate the dose, is calculated considering the patient's size, anatomic shape, and density at each position in the *z*-axis, the exposure control techniques decreases tube current for slim patients and increases tube current for obese patients.^5,20^ In this series, the correlation found between dose descriptors and BMI was even stronger, likely because all the patients with a recorded BMI underwent the CT examination on the 64-slices Optima 660 scanner that, as abovementioned, provided a mean lower radiation dose.

Despite the difference in radiation dose, comparison of objective and subjective noise values with dose descriptors did not show significant difference between the 2 scanners, thus showing that a reduction in radiation dose was not related to a lower objective image quality. The apparently skewed distribution of objective noise in CT exams performed on the 16-slice CT, is due to the absence of the ASIR. This is related to the correction loop of the ASIR within the image-generation process, which leads to significant reduction of noise independent of the dose.^21^ Indeed, the advantage of ASIR is most apparent in low dose acquisitions where noise may obscure visualization of clinically relevant information.^19^ However, the interquartile range, is narrow and concentrated on similar values for the 2 scanners, as shown by the not significant *P*-value.

In evaluation of subjective image quality, both sets of images acquired with the 2 CT scanners where rated as giving good confidence in diagnosis, with no significant differences. Images acquired on the 64-slice Optima 660 CT scanner showed a slightly significant association between dose descriptors and image noise, with a higher noise for lower doses. A significant correlation was also found between spatial resolution and TAP CTDI_vol_, while it was not found between same subjective parameter and TAP DLP. With this regard, the correlation with the CTDIvol can be considered more important, as spatial resolution refers to the plane, while DLP is related to the entire volume of acquisition. In addition, specifically on the 16-slice Lightspeed scanner, we found a strong association between dose descriptors and spatial resolution, with a higher spatial resolution for higher doses.

These results are likely related to the specific group of patients included in this cohort (ovarian cancer patients). Indeed, the subjective evaluation of images, including evaluation of image noise and spatial resolution, was focused on body structures with low mutual resolution (ie, bowel loops) in respect of possible lesions, such as peritoneal deposits. Therefore, in this specific group of patients, radiologists gave high importance to the spatial resolution, that for the ATCM techniques is more strictly related to the radiation dose,^5,22^ although the confidence in diagnosis remained good.

At the evaluation of objective and subjective image quality assessment, only the association between objective noise in aorta and confidence in diagnosis was slightly significant (*P* = 0.02), with higher confidence in diagnosis for lower objective noise. This finding is likely related to the acquisition of a single portal venous phase, acquired 70–90 seconds after the contrast medium injection, according to the indication of the radiologist performing the examination, mainly depending on the specific venous access. The use of a semi-automatic technique based on a density increase in terms of HU within a ROI placed in the lumen of the abdominal aorta would have made more homogeneous the acquisition time delay and would have probably reduced differences in subjective evaluation of the noise in the aorta. However, these techniques are typically used when arterial phases (early and/or late) are needed, such as for patients candidate to surgery, which was not the case in our cohort of ovarian cancer patients, all of which in follow-up.

There are some limitations in this study. One is that patients were included retrospectively. However, we chose a study group homogeneous for pathology (ovarian cancer) as well as for indication to undergo the CT exam (follow-up), in order to compose a homogeneous group of patients, to make comparisons consistent.

Another limitation is that it was not possible to establish the scanning extension before the CT examination. The extension may indeed vary the evaluation of the DLP and E. However, the portal venous phase included in this study, does not show many variations because, differently from other specific acquisitions, such as the pre-contrast phase or the delayed phase, that may be focused on a shorter or longer part of the body, the portal venous phase must include the whole thorax, abdomen and pelvis, despite the indication to perform the exam. Finally, as model-based IR techniques are evolving, new algorithms for reduction of radiation doses have been introduced, such as Model Based Iterative Algorithm (MBIR) proposed by GE. However, although further reductions in radiation dose are expected, these techniques are still limited by the too long reconstruction time for clinical routine, and were not evaluated in his cohort mainly because our CT machines are not equipped with them. Another limitation is that images were evaluated in consensus by 2 radiologists, without evaluation of inter-reader agreement; and subjective evaluation may vary among human observers. However, this evaluation might have been affected by the different experience of the readers (13 and 5 years of experience), and such assessment was beyond the objectives of the study.

In conclusion, current dose modulation techniques, as ASIR, may reduce radiation dose without significantly affecting image quality and diagnostic acceptability of the examinations.

These findings, that may be easily generalized from ovarian cancer patients to each patient undergoing a CT examination of thorax, abdomen and pelvis, should be taken into account when facing with the choice of a CT machine, where preference should be given to scanners with more advanced dose modulation techniques. Moreover, the differences in doses given by different MDCT machines should be taken into account in centers that own more than one CT machine, where the CT scanner better equipped for reduction of radiation dose should be considered when scanning young patients or patients who undergo frequent CT evaluations (ie, in case of follow-up examinations).
